# Expression Signatures of microRNAs and Their Targeted Pathways in the Adipose Tissue of Chickens during the Transition from Embryonic to Post-Hatch Development

**DOI:** 10.3390/genes12020196

**Published:** 2021-01-29

**Authors:** Julie A. Hicks, Hsiao-Ching Liu

**Affiliations:** Department of Animal Science, North Carolina State University, Raleigh, NC 27607, USA; jahicks3@ncsu.edu

**Keywords:** chicken, development, adipose tissue, microRNA

## Abstract

As the chick transitions from embryonic to post-hatching life, its metabolism must quickly undergo a dramatic switch in its major energy source. The chick embryo derives most of its energy from the yolk, a lipid-rich/carbohydrate-poor source. Upon hatching, the chick’s metabolism must then be able to utilize a lipid-poor/carbohydrate-rich source (feed) as its main form of energy. We recently found that a number of hepatically-expressed microRNAs (miRNAs) help facilitate this shift in metabolic processes in the chick liver, the main site of lipogenesis. While adipose tissue was initially thought to mainly serve as a lipid storage site, it is now known to carry many metabolic, endocrine, and immunological functions. Therefore, it would be expected that adipose tissue is also an important factor in the metabolic switch. To that end, we used next generation sequencing (NGS) and real-time quantitative PCR (RT-qPCR) to generate miRNome and transcriptome signatures of the adipose tissue during the transition from late embryonic to early post-hatch development. As adipose tissue is well known to produce inflammatory and other immune factors, we used SPF white leghorns to generate the initial miRNome and transcriptome signatures to minimize complications from external factors (e.g., pathogenic infections) and ensure the identification of bona fide switch-associated miRNAs and transcripts. We then examined their expression signatures in the adipose tissue of broilers (Ross 708). Using E18 embryos as representative of pre-switching metabolism and D3 chicks as a representative of post-switching metabolism, we identified a group of miRNAs which work concordantly to regulate a diverse but interconnected group of developmental, immune and metabolic processes in the adipose tissue during the metabolic switch. Network mapping suggests that during the first days post-hatch, despite the consumption of feed, the chick is still heavily reliant upon adipose tissue lipid stores for energy production, and is not yet efficiently using their new energy source for de novo lipid storage. A number of core master regulatory pathways including, circadian rhythm transcriptional regulation and growth hormone (GH) signaling, likely work in concert with miRNAs to maintain an essential balance between adipogenic, lipolytic, developmental, and immunological processes in the adipose tissue during the metabolic switch.

## 1. Introduction

During its first days post-hatch, the chick undergoes a major metabolic switch from a lipid-rich/carbohydrate-poor energy source (yolk) to a carbohydrate-rich/lipid-poor energy source (feed). Chickens, like humans, undertake lipogenesis in the liver, with little contribution from adipose tissue. As such, much effort has been undertaken to elucidate the hepatic molecular mechanisms involved in this switch, but much less is known about the role of adipose tissue. However, as it is now known that adipose tissue does not merely serve as a lipid storage site, but also carries out many endocrine functions and is also a major contributor to chronic inflammation and a number of metabolic disorders, including obesity, it is critical to understand the underlying mechanisms of adipose tissue growth and function during metabolic switching. This is of particular importance for commercial broiler breeds; as these birds have been selected for rapid growth and increased size, an unintended consequence has emerged. In addition to increased muscle mass, broilers also have excess adipose tissue accumulation and can suffer from metabolic disease (reviewed by [1]). However, it has been found that adipose tissue growth does not differ between fatter (e.g., broilers) and leaner (e.g., layers) chickens during embryogenesis and the first days post hatch, which suggests that increased rate of fat accumulation in broilers occurs later in life [2]. Therefore, it is likely that the underlying mechanisms of adipose tissue development during the switch itself are similar between these breeds and that differences in their magnitude or efficiency leads to disparities in fat accumulation.

Chickens are unique in that they only have white adipose tissue and lack the beige/brite/brown adipose tissues found in mammals [3]. Chickens have less insulin sensitivity than mammals and are naturally insulin resistant, but lack the pathologies associated with this resistance (i.e., diabetes) that are exhibited by mammals [4]. Chickens also lack orthologs of several mammalian adipokines or they are poorly conserved and have differing functions [5]. For example, the presence and function of leptin, a master regulator of feed intake (i.e., appetite) in mammals, is somewhat controversial in chickens [6]. Now, the general consensus seems to be that chickens do encode a leptin gene, however it shares little sequence homology with its mammalian counterpart and likely functions differently as it has little effect on feed intake [7]. In chickens, unlike mammals, lipolysis regulation is predominately mediated by glucagon, rather than insulin [6].

Though chicken adipose tissue has unique properties, it also shares many conserved mechanisms with mammalian adipose tissue. Vertebrate adipose tissue mass accumulates by two main mechanisms, hyperplasia (adipocyte proliferation) and hypertrophy (increase in the size of existing adipocytes due to lipid deposition). In chickens, it appears that hyperplasia dominates through the first few weeks post-hatch, after which hypertrophy dominates (reviewed by [6]). In vertebrates, the uptake and storage of serum triglycerides is mediated by very-low density lipoprotein (VLDL) (reviewed by [8]). Levels of VLDL expression are positively correlated with adiposity in chickens [9]. In addition to lower VLDL-mediated triglyceride uptake, leaner lines of chickens also have increased expression of genes associated with lipolysis [9]. Another important mediator of adipocytic lipolysis in vertebrates is the rate-limiting enzyme of triacylglycerol hydrolysis, adipose triglyceride lipase (PNPLA2, aka ATGL) [10]. *PNPLA2* KO mice have significantly larger adipocyte lipid droplets and reduced triacylglycerol breakdown, as well as reduced systemic lipid oxidation [11]. Among its many functions, growth hormone (GH) mediates lipolysis [12]. Specific knockout of growth hormone receptor (*GHR*) in mouse adipose tissue produced obese mice, due to both increased fat mass and increased adipocyte size [12]. However, *GHR* knockout did not affect the expression of most adipokines or impact glucose metabolism [12]. It has been found that that GHR regulation of lipolysis in adipocytes occurs via JAK-STAT signaling [13]. Specifically, transgenic mice unable to respond to STAT5 signaling are obese and have significantly reduced GH-stimulated lipolysis [14]. It is likely that GH signaling in chicken adipose tissue also regulates lipolysis, as *GH* polymorphisms have been linked to abdominal fat weight [15].

MicroRNAs (miRNAs) regulate a number of metabolic and immunological pathways in adipose tissue (reviewed by [16]). These include virtually all lipid metabolism, adipogenic, endocrine, and inflammatory systems. Dysregulation of miRNA adipocytic expression is often linked to the development of metabolic diseases (reviewed by [17]). This is partially due to the importance of miRNA-mediated regulation in the fate determination of mesenchymal stem cells (MSCs) [18]. In both humans and rodents, specific sets of miRNAs have been found to promote MSCs to either differentiate into osteoblasts or adipocytes (reviewed by [18]). Interference with the expression and/or function of these miRNAs can alter which cell-fate path is taken. MiRNA-mediated regulation also impacts both lipid disposition and lipolysis in adipose tissue (Reviewed by [17]). In mammals, the dysexpression of miRNAs in adipose tissue has been linked to a number of metabolic pathologies, including diabetes and obesity [19]. This suggests that adipose tissue-derived miRNAs are a vital part of the maintenance of metabolic homeostasis. For example, several profiling studies have shown that adipose tissue miRNA expression patterns differ significantly between obese and normal weight individuals (Reviewed by [20]). In vivo manipulation of miRNA functions in adipose tissue in rodent models can both exacerbate and alleviate metabolic pathologies, depending upon which miRNAs are altered [21,22,23]. In obese humans, weight loss interventions (both surgical and non-surgical) alter the adipose tissue miRNA expression profile (reviewed by [24]). It has also been discovered that mammalian adipose tissue can release miRNAs via exosomes, which can then regulate metabolic processes in other tissues, including muscle and the liver (Reviewed by [20]). Taken together, these studies highlight the diverse and vital roles miRNA have in regulating adipose-associated metabolic processes.

While a few studies have profiled miRNA in chicken adipose tissue, to our knowledge, no studies regarding the specifics of miRNA-mediated expression dynamics during the metabolic switch in adipose tissue have been published. We recently demonstrated that miRNAs regulate key hepatic metabolic pathways to help facilitate the switch [25,26]. Therefore, we undertook the present study to identify the potential involvement of adipocytically expressed miRNAs in adipose tissue development and function during the metabolic switch. To this end, we first used deep sequencing to identify differentially adipocytically expressed miRNAs and transcripts across the peri-hatching period (E18-D3) using SPF white leghorn birds. We then examined the expression patterns of a selected group of miRNAs and their targets in the adipose tissue of Ross 708 broilers during this developmental period. We found that an interconnected network of miRNAs likely works in concert to sustain lipolytic, circadian transcriptional activation and GH signaling during the embryonic to post-hatch transition.

## 2. Materials and Methods

### 2.1. Ethics Statement

All animal protocols were approved by the North Carolina State University (NCSU) Institutional Animal Care and Use Committee (IACUC#11-115-A and #17-179-A). All chick care and use procedures were performed in accordance with the United States Department of Agriculture (USDA) guidelines, using “The Guide for the Care and Use of Agricultural Animals in Research and Teaching” (FASS Ag Guide) designated by the Federation for Animal Science Societies (FASS).

### 2.2. Birds

Fertilized SPF white leghorn eggs were obtained from a commercial supplier (Charles River Laboratories, Wilmington, MA, USA) and incubated under standard conditions. At hatch, chicks were allowed ad libitum access to water and feed (Southern States chick starter feed, Richmond, VA, USA) and were brooded under standard conditions at the chicken education unit at NCSU. At E18, E20, D0 (after hatching/prior to feeding), D1, and D3, chicks were humanely euthanized by cervical dislocation and abdominal adipose tissue was collected and snap frozen in liquid nitrogen and stored at −80 °C until analysis. Fertilized Ross 708 eggs were obtained from the NCSU chicken education unit and incubated under standard conditions. At hatch, chicks were feather sexed (sex was confirmed by examination of the gonads at time of tissue collection for all time points) and weighed and were brooded under standard conditions at the chicken education unit at NCSU. Males weighing between 46 g and 48 g were used for further analysis and received water and chick starter feed (Southern States) ad libitum on day of hatch. At E18, E20, D0 (after hatching/prior to feeding), D1, and D3, chicks were humanely euthanized by cervical dislocation and abdominal adipose tissue was collected and snap frozen in liquid nitrogen and stored at −80 °C until analysis.

### 2.3. Next Generation Sequencing Library Construction

Total RNA was isolated using Tri-Reagent (Sigma-Aldrich, St. Louis, MO, USA) following the manufacturer’s instructions, with the modification of precipitation of the RNA overnight at −20 °C. RNA quality was accessed using an Agilent 2100 Bioanalyzer (Santa Clara, CA, USA) with a high sensitivity RNA chip. All RNA samples had RIN > 9.

Small RNAs were enriched from total RNA using a miRVana kit (Thermo Fisher Scientific, Waltham, MA, USA) following the manufacturer’s instructions. To produce the small RNA NGS libraries, enriched small RNAs for each timepoint were pooled equally from four birds and one microgram of pooled enriched small RNAs were used per library. Libraries were generated using a TruSeq small RNA library preparation kit (Illumina, San Diego, CA, USA) and Illumina barcode primers for multiplexing, following the manufacturer’s instructions. Library quantity and quality were assessed on an Agilent 2100 Bioanalyzer with a high sensitivity DNA chip. Equal molar amounts of the libraries were pooled and submitted to the Genomics Science Laboratory at NCSU and sequenced on a single lane (35bp-single end) of an Illumina Genome Analyzer IIx.

For RNA-seq library construction, a TruSeq RNA library preparation kit v2 and barcode indices for multiplexing were used, following the manufacturer’s instructions (Illumina). For each developmental timepoint, individual libraries were produced from four individual birds (*n* = 4) using one microgram of total RNA per library. Library quantity and quality were determined using an Agilent Technologies high sensitivity DNA chip on a 2100 Bioanalyzer. Equal molar amounts of the libraries were pooled and 50bp-single end sequenced using an Illumina HiSeq2500 at DHMRI (Kannapolis, NC, USA).

### 2.4. Next Generation Sequencing Analysis

FASTQ files are available in the NIH Short Read Archive (Bioproject PRJNA600484: https://www.ncbi.nlm.nih.gov/sra/PRJNA600484). All sequencing data processing and analyses were performed using CLC genomics workbench (Qiagen, Germantown, MD, USA). The NGS trim tool was used to remove any residual adaptor sequences and/or low quality sequences (Phred < 20). Reads were mapped to the *Gallus gallus* reference genome (Gallus_gallus-6.0) and normalized using the transformation and normalization tool. Expression analysis of the small RNA libraries was carried out using the small RNA analysis suite, and mRNA libraries were analyzed using the RNA-seq analysis suite. Specifically, differential expression was determined using the “Empirical analysis of DGE” tool, which implements the “exact test” developed by Robinson and Smyth and a FDR corrected *p*-value cutoff of 0.05. Pairwise comparisons were made between developmental time points. An ingenuity pathway analysis (Qiagen) was then performed on the differential expression data using the integrated plugin.

### 2.5. Real-Time Quantitive PCR (RT-qPCR)

Total RNA was isolated using Tri-Reagent (Sigma-Aldrich) following the manufacturer’s instructions, with the modification of precipitation of the RNA overnight at −20 °C and DNase-treated using a TURBO-DNA free kit as directed (Thermo Fisher Scientific). At each timepoint, six individual male birds (Ross 708) were used for RT-qPCR (*n* = 6). RNA quality was assessed using agarose electrophoresis. One microgram of DNase-treated total RNA per sample was reverse transcribed using a miScript II RT kit (Qiagen) following the manufacturer’s instructions.

Primers used for RT-qPCR are provided in Supplementary Materials Table S1. Primers used for miRNA quantification consisted of the mature miRNA sequence (obtained from miRBase: http://www.mirbase.org/) for the forward primer and the universal reverse primer provided by Qiagen. For mRNA quantification, forward and reverse primers were designed using primer-BLAST (https://www.ncbi.nlm.nih.gov/tools/primer-blast/), and when possible, such that one of the primers spanned an exon-exon junction. For miRNA quantification, 5 ng of cDNA was used per reaction and for mRNA quantification 10 ng cDNA were used. Ribosomal protein S24 (*RPS24*) and small nucleolar protein U83B (*snoU83B*) were used as housekeeping genes for mRNA and miRNA expression normalization, respectively. Gene/miRNA-specific primers were used at a final concentration of 500 nM and the Qiagen universal reverse primer was used at a concentration of 1X. RT-qPCR reactions also contained 1X iQ SYBR Green Supermix (Bio-Rad, Hercules, CA, USA). RT-qPCR conditions were as follows: 95 °C for 5 min, followed by 40 cycles of 95 °C for 10 s, then 58 °C for 20 s. All reactions were performed in duplicate. Melting curve analysis was utilized to confirm gene-specific amplification. Significant differences (*p* < 0.05) in expression were determined using analysis of variance.

## 3. Results

### 3.1. Small RNA-Seq and RNA-Seq Library Characteristics

The number of trimmed mappable, high quality (phred ≥ 20) reads in the small RNA libraries ranged from 641,971–1,266,436. The number of trimmed mappable, high quality (phred ≥ 20) reads in the mRNA libraries ranged from 6,802,329–14,678,927. The number of unique miRNAs in each library (CPM ≥ 30) ranged from 82–92. Identified miRNAs are listed in Table S2. The number of unique transcripts (RPKM ≥ 30) ranged from 4011 to 4433. Identified transcripts are listed in Table S3.

### 3.2. Expression Dynamics of miRNAs in Adipose Tissue during the Peri-Hatching Period in Chickens

Pairwise comparisons between the small RNA adipose tissue libraries generated from white leghorns were used to first examine miRNA expression dynamics across the peri-hatching period. Between E18 and E20, 15 miRNAs were upregulated and seven miRNAs were downregulated. In the E20 and D0 comparison, 18 miRNAs were higher expressed in the adipose tissue of D0 birds and nine miRNAs were lower expressed. Between D0 and D1, 20 miRNAs were upregulated and 13 were downregulated. Comparison of the D1 and D3 miRNA profiles of adipose tissue identified 24 upregulated miRNAs and 10 downregulated miRNAs. Post-switch (D3) adipose tissue had 63 upregulated miRNAs compared to pre-switch (E18) adipose tissue and 30 downregulated miRNAs. At all timepoints, the highest expressed miRNA in the adipose tissue was *miR-10b*.

### 3.3. Modulation of the Adipocytic Transcriptome during the Metabolic Switch in Chickens

From E18 to E20, the expression of 505 genes was significantly (≥2-fold; FDR corrected *p*-value ≤ 0.05) altered in the adipose tissue of white leghorn embryos, with 238 genes upregulated and 267 genes downregulated. Comparison of the E20 and D0 adipose tissue transcriptomes identified 202 differentially (≥2-fold; FDR corrected *p*-value ≤ 0.05) expressed genes; of these, 105 were upregulated and 97 were downregulated. Between D0 and D1, 125 genes had expression differences (≥2-fold; FDR corrected *p*-value ≤ 0.05) in the adipose tissue, with 43 upregulated genes and 82 downregulated genes. During the D1 to D3 developmental phase, 148 genes were found to have altered expression (≥2-fold; FDR corrected *p*-value ≤ 0.05), with 60 upregulated genes and 88 downregulated genes. During the metabolic switch which occurs in the course of the embryonic (E18) to post-hatch (D3) transition, 467 genes were identified with significantly different (≥2-fold; FDR corrected *p*-value ≤ 0.05) expression levels, with 183 genes upregulated and 284 genes downregulated.

**Figure 1:**
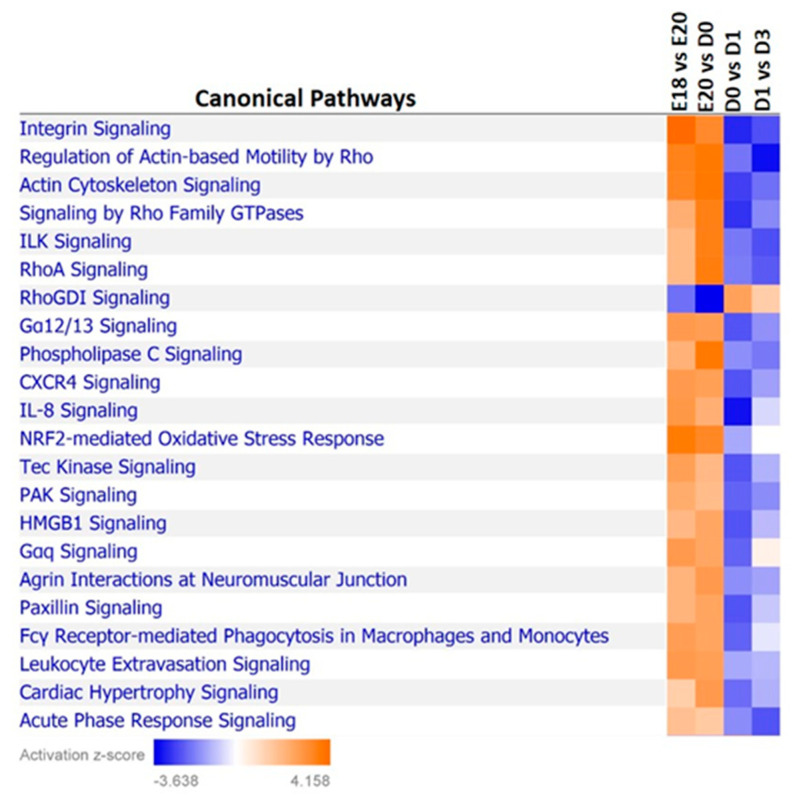
Comparison of canonical pathway activation z-scores in the adipose tissue of pre- and post-hatch chicks. Ingenuity Pathway Analysis was utilized to compare the gene expression signatures of canonical pathways identified in the transcriptome profiling of the adipose tissue (white leghorn) in late stage embryos and early post-hatch chicks. Many canonical pathways have a defined demarcation in their predicted activation/inhibition (z-score) at the time of hatch (D0).

Interestingly, ingenuity pathway analysis (IPA) of the transcriptome data revealed a number of canonical pathways which showed a distinct demarcation across the embryonic to hatch transition in their likelihood of activation or inhibition (Figure 1). These include several cytoskeleton-associated pathways and inflammation-associated pathways (Figure 1). A number of cellular growth and survival, lipid metabolism, oxidative stress and cell cycle-associated pathways were among the top most affected cellular pathways in adipose tissue during the metabolic switch (Table 1).

### 3.4. Expression Patterns of miRNA: Target Gene Pairings in Adipose Tissue during the Metabolic Switch

**Figure 2:**
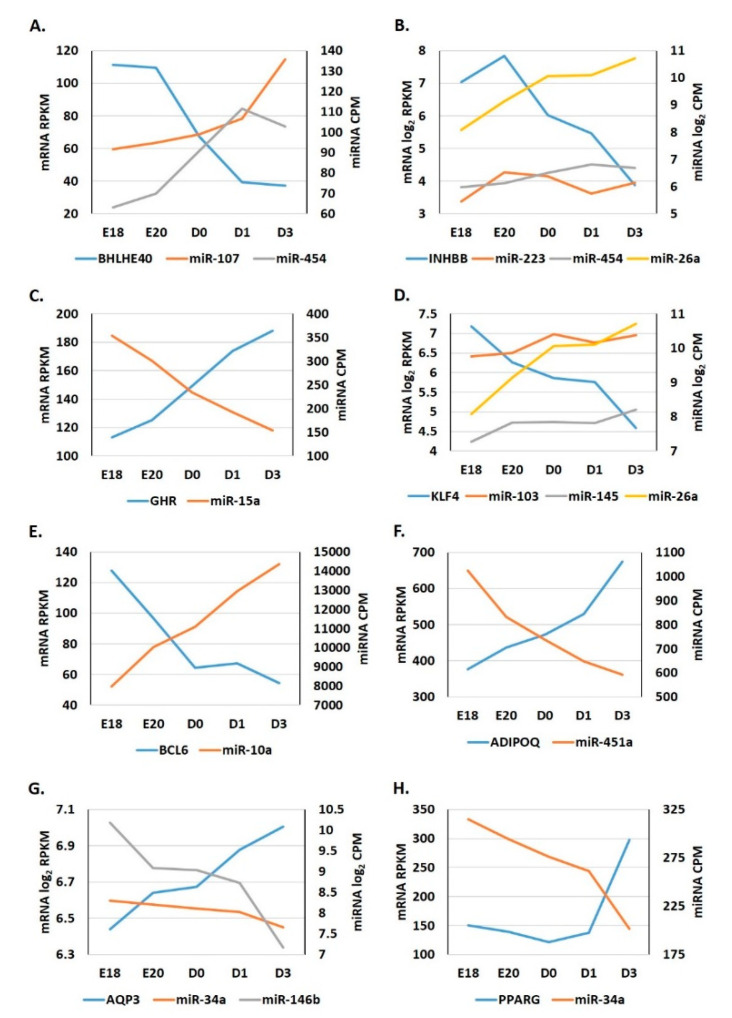
(**A**) Representative selection of miRNA:target gene adipocytic expression signatures in peri-hatch white leghorn chicks. The expression profiles of miRNAs and gene transcripts in the adipose tissue of E18, E20, D0, D1, and D3 chicks were determined using small RNA-seq and RNA-seq analyses. A. Expression of *BHLH40* and its targeting miRNAs, *miR-107* and *miR-454*. (**B**) Expression of *INHBB* and its targeting miRNAs, *miR-223*, *miR-454*, and *miR-26a*. (**C**) Expression of *GHR* and its targeting miRNA, *miR-15a*. (**D**) Expression of *KLF4* and its targeting miRNAs, *miR-103*, *miR-145*, and *miR-26a*. (**E**) Expression of *BCL6* and its targeting miRNA, *miR-10a*. (**F**) Expression of *ADIPOQ* and its targeting miRNA, *miR-451a*. (**G**) Expression of *AQP3* and its targeting miRNAs, *miR-34a* and *miR-146b*. (**H**) Expression of *PPARG* and its targeting miRNA, *miR-34a*. Data are presented as normalized expression values: RPKM for mRNA and CPM for miRNA.

Utilization of the miRNA and mRNA next generation sequencing profiles in conjunction with the microRNA Target Filter of IPA identified a number of miRNA which likely contribute to the regulation of adipose tissue-associated genes and pathways during the metabolic switch. These include the circadian rhythm inhibitor *BHLHE40* and its targeting miRNAs, *miR-107* and *miR-454*. *BHLHE40* expression was significantly lower in the adipose of D3 birds than in E18 birds (Figure 2A), while *miR-107* and *miR-454* had higher adipocytic expression at D3 than E18. *MiR-454* also had reciprocal adipocytic expression with another of its targets *INHBB* (Figure 2B).

Growth hormone receptor (*GHR*) expression in the adipose tissue steadily increased during embryonic to hatch transition, while its regulating miRNA *miR-15a* steadily declined (Figure 2C). The adipocytic expression of *KLF4*, a major transcriptional regulator of a variety of embryonic developmental processes, also decreased after hatching, while the expression of its targeting miRNAs, *miR-103*, *miR-145*, and *miR-26a*, tended to increase post-hatching (Figure 2D). The expression of the transcriptional repressor *BCL6* quickly declined after hatching and the expression of *miR-10a*, a *BCL6* regulator, continually increased from E18 to D3 (Figure 2E). *ADIPOQ*, one of the core adipokines responsible for the regulation of many metabolic pathways in both adipose and non-adipose tissues, had significantly higher expression in the adipose tissue of D3 birds than in E18 birds, *miR-451a*, which regulates *ADIPOQ* expression, had lower adipocytic expression post-hatching (Figure 2F). The expression of *AQP3*, a member of the aquaporin family of water channels which is associated with glycerol permeability, increased post-hatching in adipose tissue, while the expression of two of its potential miRNA regulators, *miR-34a* and *miR-146b*, tended to decline (Figure 2G). *MiR-34a* also likely regulates *PPARG* expression in adipose tissue, which tended to increase post-hatching (Figure 2H).

### 3.5. Expression Patterns of miRNA: Target Pairings in the Adipose Tissue of Ross 708 Broilers

**Figure 3:**
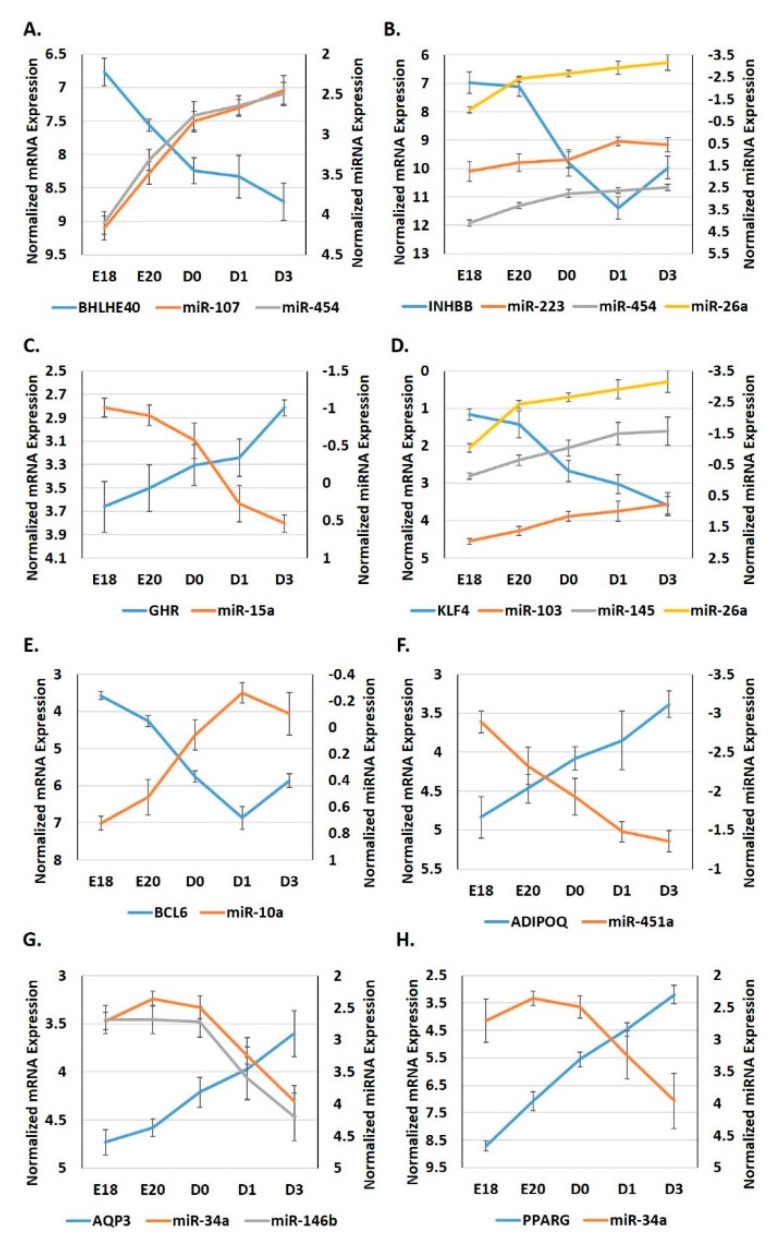
RT-qPCR adipocytic expression profiling of selected miRNAs and their target genes in Ross 708 broilers. The expression signatures of miRNA/target pairings of interest, identified in the NGS study, were determined in the adipose tissue of Ross 708 broilers using RT-qPCR. (**A**) Expression of *BHLH40* and its targeting miRNAs, *miR-107* and *miR-454*. (**B**) Expression of *INHBB* and its targeting miRNAs, *miR-223*, *miR-454*, and *miR-26a*. (**C**) Expression of *GHR* and its targeting miRNA, *miR-15a*. (**D**) Expression of *KLF4* and its targeting miRNAs, *miR-103*, *miR-145*, and *miR-26a*. (**E**) Expression of *BCL6* and its targeting miRNA, *miR-10a*. (**F**) Expression of *ADIPOQ* and its targeting miRNA, *miR-451a*. (**G**) Expression of *AQP3* and its targeting miRNAs, *miR-34a* and *miR-146b*. (**H**) Expression of *PPARG* and its targeting miRNA, *miR-34a*. Data are presented as normalized expression values (ΔCt).

We employed RT-qPCR to examine the expression patterns of miRNA:target pairings of interest during the metabolic switch in the adipose tissue of a commercial broiler breed, Ross 708 (Figure 3).

We found that overall, these miRNAs and their targets had similar adipocytic expression signatures, between white leghorns and Ross 708 chickens, i.e., similar directional changes over the course of the metabolic switch (Figure 3). The expression of *BHLHE40* significantly (*p* < 0.05) decreased 3.8 fold in the adipose tissue of Ross 708 birds from E18 to D3, while the expression of its targeting miRNAs, *miR-107* and *miR-454* both increased (*p* < 0.05) approximately 3-fold during this period (Figure 3A). There was an 8-fold (*p* < 0.05) decrease in *INHBB* adipocytic expression from E18 to D3; conversely, there was a significant (*p* < 0.05) increase in the expression of its targeting miRNAs *miR-454* (~3-fold) and *miR-26a* (4.4-fold) (Figure 3B). Another *INHBB* targeting *miR-223* had increased expression (~2-fold) from E18 to D3, but was not statistically significant (*p* > 0.05). Adipocytic expression of GHR increased moderately from E18 to D3 (1.8-fold; *p* < 0.05), while its targeting miRNA, *miR-15a*, decreased ~3-fold (*p* < 0.05) during this period (Figure 3C). The expression of *KLF4* decreased ~5-fold (*p* < 0.05) in Ross 708 adipose tissue from E18 to D3, whereas the expression of three *KLF4* regulators, *miR-103*, *miR-145* and *miR-26a*, significantly (*p* < 0.05) increased from about 2- to 4-fold (Figure 3D). From E18 to D3, the expression of *BLC6* in adipose tissue decreased approximately 5-fold (*p* < 0.05), while *miR-10a* expression, a *BCL6* regulator, increased about 2-fold (*p* < 0.05) (Figure 3E). *ADIPOQ* was expressed 2.7-fold (*p* < 0.05) higher in the adipose tissue of Ross 708 chicks compared to that of E18 embryos (Figure 3F). The *ADIPOQ* targeting miRNA, *miR-451a* was expressed 2.9-fold (*p* < 0.05) lower in the adipose tissue between D3 and E18 (Figure 3F). *AQP3* adipocytic expression was slightly over 2-fold (*p* < 0.05) higher at D3 than E18, whereas two of its miRNA regulators, *miR-34a* and *miR-146b*, were both significantly (*p* < 0.05) lower, with a 2.4-fold and 2.9-fold decrease, respectively (Figure 3G). *MiR-34a* also targets *PPARG*, which significantly increased in the adipose tissue after hatching (Figure 3H).

### 3.6. Examination of miRNA-Regulated Networks in Adipose Tissue during the Metabolic Switch

**Figure 4:**
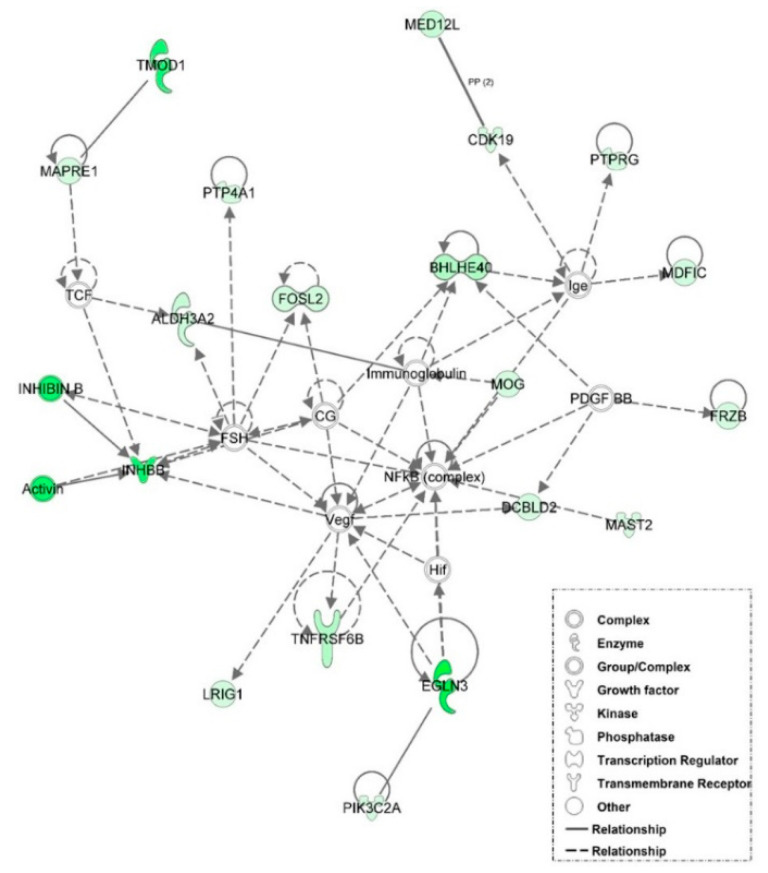
Network mapping of *miR-454* regulated genes. The adipocytic expression of miR-454 is upregulated at D3 compared to E18. A number of *miR-454* target genes associated with cellular assembly and organization, lipid metabolism, and the circadian rhythm were downregulated in the adipose tissue of D3 birds relative to E18 birds in the RNA-seq data. Green molecules had significantly (*p* < 0.05) lower expression at D3. Solid lines indicate a direct interaction and dashed lines indicate an indirect interaction.

**Figure 5:**
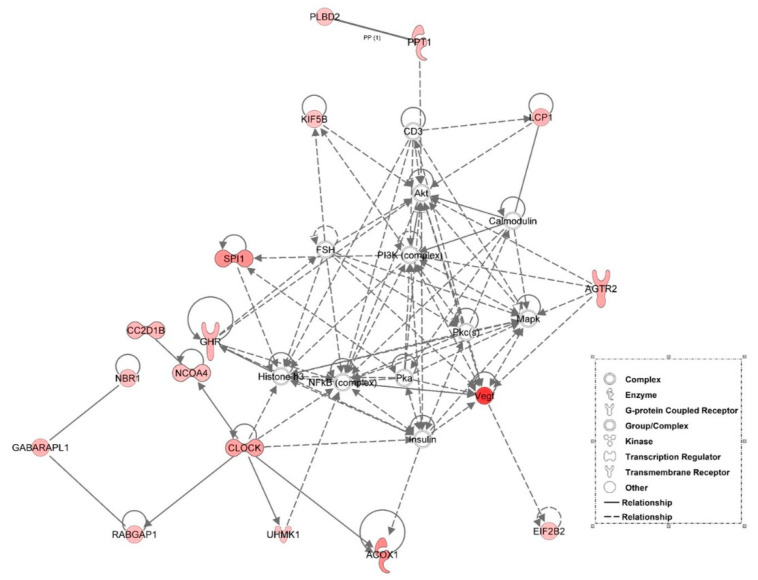
Network mapping of *miR-15a* target genes. From E18 to D3, the adipocytic expression of miR-15a declined. Network mapping of *miR-15a* upregulated (from E18 to D3) target genes in the adipocytic transcriptome data, identifying a group of targets associated with organismal development and the circadian rhythm. Red molecules had significantly (*p* < 0.05) higher expression at D3. Solid lines indicate a direct interaction and dashed lines indicate an indirect interaction.

**Figure 6:**
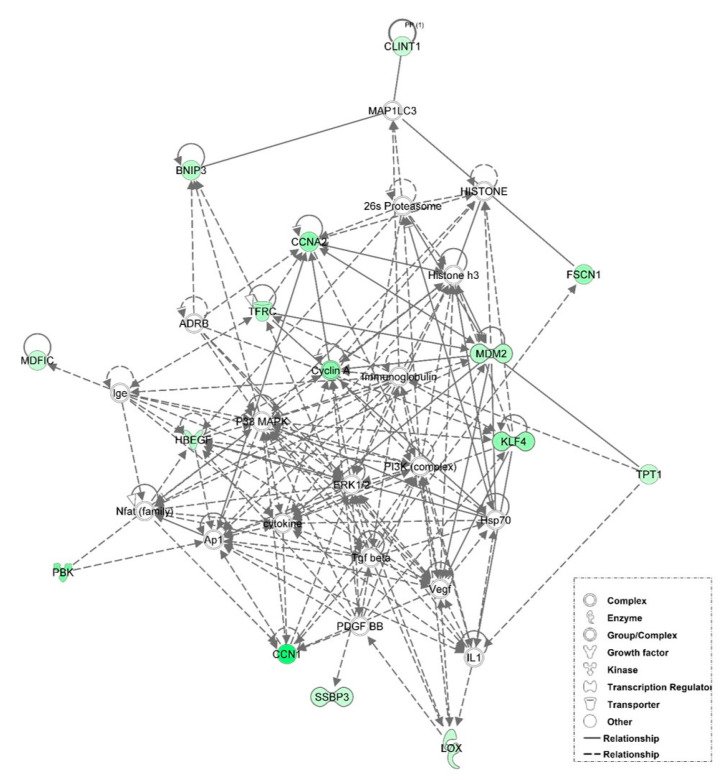
Network mapping of *miR-145* target genes. *MiR-145* expression in the adipose tissue increased from E18 to D3. MiR-145 target genes found to be downregulated during this period include those associated with cellular development, cellular growth and proliferation and cellular movement. Green molecules were significantly (*p* < 0.05) downregulated from E18 to D3. Solid lines indicate a direct interaction and dashed lines indicate an indirect interaction.

**Figure 7:**
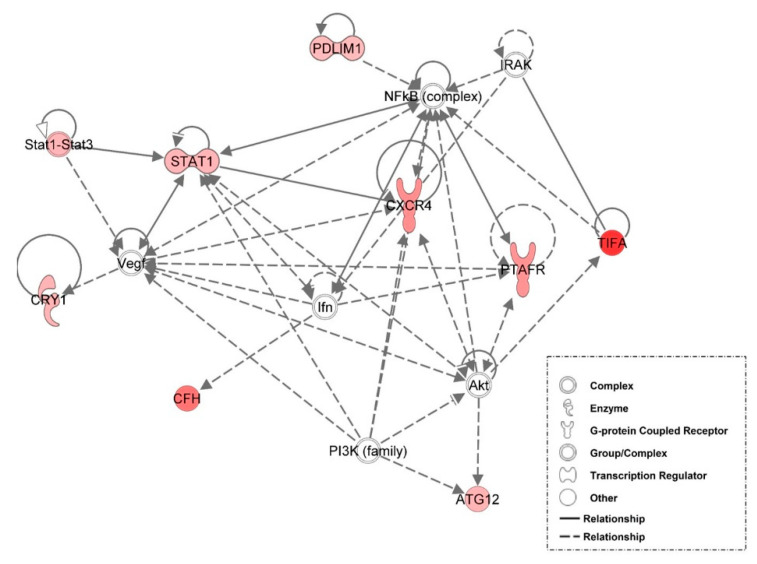
Network mapping of *miR-146b* target genes. There was a decrease in *miR-146b* adipocytic expression, from E18 to D3. Several *miR-146b* target genes involved in cellular movement and immune cell trafficking were upregulated during this time. Red molecules had significantly (*p* < 0.05) higher expression in D3 adipose tissue. Solid lines indicate a direct interaction and dashed lines indicate an indirect interaction.

**Figure 8:**
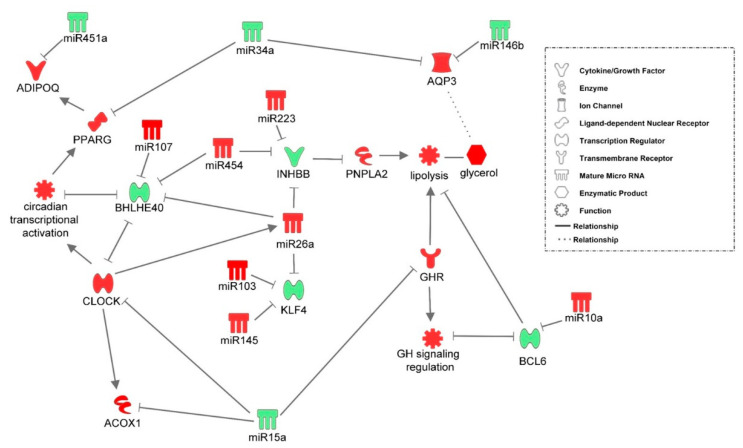
An adipocytic transcriptional and post-transcriptional regulatory system identified in chicks during the embryonic-hatch transition. A complex network of transcriptional regulators and miRNAs likely work in concert to control adipogenic and lipolytic processes during the shift from embryonic metabolism to the post-hatching metabolism. Red molecules have higher expression in D3 adipose tissue than E18 adipose tissue and green molecules have lower expression. Gears represent biological processes and their expected expression pattern is based on the expression signatures of the genes controlling them.

Further examination of miRNA regulatory networks identified in the NGS study revealed that metabolic switch-associated adipocytic miRNAs likely regulate a number of important interconnected developmental and metabolic gene networks (Figure 4, Figure 5, Figure 6, Figure 7 and Figure 8). As discussed above, *miR-454* is upregulated from E18 to D3 in the adipose tissue of both layers and broilers (Figure 2 and Figure 3). The network mapping of *miR-454* target genes, which were found to be significantly downregulated from E18 to D3 in the adipocytic transcriptomes generated in the NGS study, suggests that it could potentially regulate cellular assembly and organization, lipid metabolism, and circadian rhythm processes during metabolic switch (Figure 4). *MiR-15a*, which was found to be significantly downregulated from E18 to D3 (Figure 2 and Figure 3), also may regulate developmental, lipid metabolism and circadian rhythm networks (Figure 5). We found that *miR-145* adipocytic expression significantly increases from E18 to D3 (Figure 2 and Figure 3), and network mapping of downregulated *miR-145* targets during this period, suggests that it is involved in regulating cellular development, growth and proliferation (Figure 6). Downregulation of adipocytic expression of *miR-146b* from E18 to D3 (Figure 2 and Figure 3) likely modulates immune cell trafficking (Figure 7). Taken together, these results suggest a complex regulatory model, in which a subset of miRNAs work concordantly to regulate a diverse group of developmental, immune and metabolic processes, in the adipose tissue during the metabolic switch in chickens (Figure 8).

## 4. Discussion

Chickens, like humans, synthesize lipids in the liver which are, in times of caloric excess, stored in adipose tissue. However, chickens are unique in that they only generate white adipose tissue and lack the beige/brown adipose tissues found in mammals. Chickens also lack several key metabolic hormones found in other vertebrates, including leptin, resistin, and omentin [5]. Chickens are also much less sensitive to insulin and can tolerate plasma glucose levels that would be fatal in mammals. Furthermore, during the peri-hatch period, chicks naturally undergo an acute metabolic shift from a mainly lipolytic (energy derived from lipid-rich yolk) to a lipogenic (energy source is lipid-low/carbohydrate-rich feed) state within a very short period of time.

Though the liver is the main site for lipid production in birds and humans, it has been well established that adipose tissue is more than a mere lipid storage site and carries out many important endocrine and immune functions. We recently demonstrated that miRNAs regulate many hepatic metabolic pathways during the metabolic transition of neonatal chicks [25,26]. Here, we expand this analysis of metabolic miRNA by examining miRNA expression dynamics in the adipose tissue during the peri-hatching period. Though the magnitude of metabolic gene expression can differ between commercial layers (e.g., white leghorn), which have a leaner phenotype and commercial broilers (e.g., Ross 708), which have a fatter phenotype, the overall contributing metabolic processes and pathways are similar [9]. Additionally, these processes have been found to differ little between fatter and leaner chicks during embryogenesis and the first days post-hatch [2]. As adipose tissue is well known to produce inflammatory and other immune factors [27], we used SPF white leghorns to generate the initial miRNome and transcriptome signatures to minimize complications from external factors (e.g., pathogenic infections) and ensure the identification of bona fide switch-associated miRNAs and transcripts. We then examined their expression signatures in the adipose tissue of broilers (Ross 708) during this developmental time period. Overall, these miRNAs and their targets had similar adipocytic expression signatures, between white leghorns and Ross 708 chickens (Figure 2 and Figure 3), suggesting that they are part of a conserved core regulatory system which governs energy expenditures and lipid storage during the metabolic reprogramming period.

Transcriptome and miRNome analyses revealed that a dynamic and diverse metabolic program exists in the adipose tissue during the embryonic-hatch transition in chicks. Several important developmental and metabolic systems have distinct expression patterns between adipose tissue of embryonic and post-hatch chicks (Figure 1). For example, signaling by Rho GTPases, particularly, RhoA signaling is likely activated in adipose tissue of late stage embryos compared to post-hatch chicks; conversely, inhibitors of Rho GTPases, specifically RhoGDI signaling is inhibited in adipose tissue of late stage embryos compared to post-hatch chicks. Rho signaling is involved in a variety of cellular processes including, cytoskeleton organization and actin polymerization (reviewed by [28]). Two major processes, hypertrophy (de novo adipocyte production) and hyperplasia (increased lipid accumulation in existing adipocytes) contribute to increased adipose tissue mass. In mice, it has been shown that the physical stretching of adipocytes due to increased lipid accumulation increases Rho and Rho kinase signaling [29]. Mice expressing a dominate-negative form of *RhoA* in adipocytes exhibited fewer deleterious effects from a high fat diet including, reduced weight gain, reduced macrophage recruitment and inflammation and reduced hypertrophy [29]. If RhoA signaling functions similarly in chicken adipose tissue, then higher Rho activity in chicken embryonic adipose tissue may be due to the increased yolk lipid uptake and storage (i.e., hypertrophy) that occurs during the last days of embryonic development (reviewed by [6]). This is further supported by lower expression in lipolytic factors in the adipose tissue of E18 embryos compared to D3 chicks (Figure 2, Figure 3 and Figure 8), which also indicates that there are likely larger amounts of lipid stores in E18 embryos and that D3 chicks are still heavily reliant upon adipose tissue lipid stores for energy production, and are not yet efficiently using their new food source for de novo lipid production and storage. 

The adipocytic transcriptomes and miRNomes presented here suggest that a select group of miRNAs work concertedly to increase lipolysis and restrict adipogenesis during the peri-hatching period. For example, the adipocytic expression of *miR-26a* increases from E18 to D3 (Figure 2 and Figure 3). *MiR-26a* is known to target a number of metabolic and adipogenic genes (reviewed [30]). Activins are members of the TGF-β superfamily which are highly expressed in adipose tissue [31]. Activin B is comprised of an InhibinβB (INHBB) homodimer. Activin B was shown to decrease lipolytic activity and increase intracellular triglyceride storage in the adipocyte cell line, 3T3-L1 [31]. SiRNA-mediated INHBB knockdown in these cells resulted in increased expression of adipose triglyceride lipase (PNPLA2; rate-limiting enzyme in triglyceride hydrolysis), while overexpression of Activin B in 3T3-L1 cells reduced PNPLA2 expression and suggests that its lipolytic inhibitory effects are concentration dependent [31]. In the present study, adipocytic expression of *INHBB* was down-regulated -7.73-fold from E18 to D3 (Figure 2B), while *PNPLA2* was up-regulated 5.51-fold during this transition (Table S3). A conserved *miR-26a* (up-regulated 6.18-fold from E18-D3) putative target is *INHBB* (www.targetscan.org). It is possible that the increased adipocytic expression of *miR-26a* during the embryonic to hatch transition serves, in part, to relieve Activin B inhibition of *PNPLA2* expression to facilitate the lipolysis of adipose tissue triglyceride stores to supply the energy needed for hatching and the rapid growth which follows. *MiR-26a* also targets a member of the kruppel-like factor family, *KLF4* [32]. KLF4 was shown to be a critical inducer of adipogenesis, at least in part, by activating CEBPB expression [33]. During adipogenesis, CEBPB is important for maintaining a proliferative state, as the reduction of CEBPB induces the expression of a number of adipogenic differentiating factors leading to adipocytic differentiation [34]. Thus, it is possible that one function of adipose-expressed *miR-26a* may be to promote the utilization of already established lipid stores to provide the energy necessary for hatching and concurrently limiting the production of new adipocytes hyperplasia) during this time of high energy need.

In mice, PNPLA2-induced acute lipolysis leads to increased infiltration and activation of immune cells into adipose tissue [11]. The oxidative stresses associated with increased fatty acid oxidation will likely impact the immunological status of adipose tissue during this time. It has been well established in mammals that obesity leads to chronic inflammation in adipose tissues, which causes a number of deleterious effects (reviewed by [35]). In turn, weight loss and fasting typically induce anti-inflammatory immune molecules in adipose tissue (reviewed by [35]). This induction of anti-inflammation is accompanied by a phenotypic switch of adipose-associated macrophages from the M1 to the M2 state (reviewed by [36]). The major adipokine adiponectin (ADIPOQ) plays a myriad of roles, including modulation of adipocytic immunological responses, for example, promoting anti-inflammatory cytokines and inhibiting pro-inflammatory cytokines [37]. In chickens, ADIPOQ levels were also shown to be negatively correlated with lipid disposition in adipose tissue and positively correlated with PNPLA2 levels [38]. Here, *ADIPOQ* levels were up-regulated in post-hatch chick adipose tissue, again, suggesting that during the peri-hatching period, utilization of fat stores for energy production takes precedent over de novo storage of lipids. This up-regulation of *ADIPOQ* is likely due, partially, at the post-transcriptional level, to reduced expression of *miR-451a*, an *ADIPOQ* regulator (www.targetscan.org). At the transcriptional level, increased *ADIPOQ* may also be due to increased PPARγ (PPARG) levels, a transcriptional inducer of *ADIPOQ*, as adipocytic expression of *PPARG* was also significantly higher in post-hatch vs. embryonic chicks (Figure 2, Figure 3 and Figure 8). Increased *PPARG* levels in turn may be due to decreased expression of targeting miRNAs, particularly *miR-34a*, which is down-regulated in post-hatch adipose tissue (Figure 2, Figure 3 and Figure 8). As discussed above, ADIPOQ induces a more anti-inflammatory state in adipose tissue. Further supporting a more anti-inflammatory state in D3 adipose tissue compared to E18 adipose tissue is the predicted activation of CXCR4 signaling before hatching and inhibition after hatching (Figure 1). CXCR4 signaling is positively correlated to a pro-inflammatory state [39]. IL8 signaling is also predicted to be activated in the adipose tissue of pre-hatch embryos and inhibited in that of post-hatch chicks (Figure 1). IL8 (aka CXCL8) is considered to be an important mediator of inflammation in its role as a chemotactic chemokine for and activator of a variety of immune cells (reviewed by [40]). Together, these results suggest that lipid accumulation in late stage chick embryos likely induces a pro-inflammatory state in adipose tissue and that the rapid utilization of these lipid stores in the first days post-hatching relieves this inflammation. They also support a role for miRNA-mediated regulation of important mediators of inflammation in adipose tissue, e.g., ADIPOQ, in the modulation of inflammatory dynamics of adipose tissue during the metabolic switch.

In recent years, it has been established that the circadian rhythm and metabolic processes are interconnected (reviewed by [41]). A central clock located in the SCN (suprachiasmatic nuclei) of the hypothalamus serves as a master regulator of peripheral clocks located throughout the body, including adipose tissues [42]. The main core clock proteins are CLOCK (1.96-fold increase from E18 to D3, *p* < 0.05) and BMAL1 which function in autoregulatory-feedback loops to control both their own expression as well as that of many other genes [43]. In adipose tissue, the circadian rhythm regulates a variety of metabolic processes including adipogenesis, lipolysis, and lipid storage (reviewed by [44]). The clock can regulate metabolic enzymatic activity to compensate for varying energy demands during the circadian period, i.e., higher demands during the active period and lower demands during the rest period. It can also modulate these metabolic processes in response to feed intake, i.e., the fed state (active) and fasted state (resting) (reviewed by [41]). As such, disruptions in circadian clock regulation have been linked to the development of metabolic disorders, such as an increased risk of obesity [45]. Another branch of the clock autoregulatory-feedback loop is *BHLHE40* (3.02-fold decrease from E18 to D3, *p* < 0.05). BHLHE40 is a transcriptional repressor of CLOCK and therefore CLOCK-controlled genes [43]. It also regulates its own transcription. BHLHE40 was found to indirectly repress *PPARG* expression by interacting with CEBPs [43]. *BHLHE40* KO mice have a disrupted circadian rhythm, with a longer circadian period than WT mice [43]. These KO mice also have lower amounts of lipid disposition and suppressed adipocyte differentiation in white adipose tissue. In the present study, we found that *BHLHE40* expression was significantly lower in adipose tissue of post-hatch chicks than embryonic chicks (Figure 2, Figure 3 and Figure 8). The increased expression of two *BHLHE40*-regulating miRNAs, *miR-107* and *miR-454* (Figure 2, Figure 3 and Figure 8), in the adipose of post-hatch chicks is a likely contributing factor to the down-regulation of *BHLHE40* expression. These results suggest that the circadian clock is a key regulator of adipogenic and lipolytic processes in the adipose tissue during the embryonic to hatch transition in chickens. These results also indicate that the circadian clock is activated post-hatching in the adipose tissue, likely to increase the utilization of adipocytic lipid stores to meet the higher energy demands associated with hatching itself, as well as the increased activity of the chick. It also appears that adipocytically expressed miRNAs regulate the major factors of the circadian clock (Figure 8). It has been demonstrated that disruption of the diurnal period in chickens affects adipose tissue lipid deposition and bird weight [46]. Broilers exposed to varying periods of light differed significantly in adipose accumulation and consequently body weight. It was found that birds exposed to both shorter and longer light periods had significantly less adipose tissue than birds exposed to 18–22 h of light [46]. This suggests that chicken adipogenic/metabolic processes are under circadian regulation, in a similar fashion as that of mammals. These observations further support a role for circadian rhythmic genes in the balance of lipid storage and lipid utilization during the embryonic to hatch metabolic transition of the chick.

Growth hormone (GH) regulates many developmental and metabolic processes, including increased lipolysis (reviewed by [12]). GH is predominantly produced in the brain and then released system-wide to regulate these processes. Here, we found that the adipocytic expression of the GH receptor (*GHR*) significantly increases (2-fold, *p* < 0.05) during the embryonic-hatch transition. We further found that this increase in *GHR* may be potentially due in part, to the reduced (2.29-fold) expression of *miR-15a* (a *GHR* regulator) during this time. GH signaling can inhibit the expression of *BCL6* and in turn BCL6 is able to repress GH-regulated transcription [47]. BCL6, though initially found to be a repressor of B-cell responses, is now known to be expressed in a wide variety of tissues and functions as a transcriptional repressor in many cellular pathways [48]. *BCL6* KO mice exhibit abnormal lipid metabolism and it was suggested that in adipose tissue BCL6 aids in the regulation of adipogenesis [48]. It is also possible that BCL6 is a positive regulator of adipose tissue hypertrophy and/or a negative regulator of lipolysis [48]. In the present study, we found that *BCL6* is down-regulated (-1.91-fold, *p* < 0.05) during the peri-hatching period while, its miRNA regulator, *miR-10a* is up-regulated (1.8-fold). IGFBP5 is a member of the insulin-like growth factor family, which is stimulated by GH signaling [49]. In a mouse myoblast cell line, IGFBP5 was found to both decrease lipid accumulation and also promote lipolysis [50]. Here, we found that adipocytic expression of *IGFBP5* was up-regulated from E18 to D3 (2.52-fold higher, *p* < 0.05) and conversely the *IGFBP5* regulator, *miR-140*, was down-regulated (-2.12-fold) during this time. Taken together these results suggest that miRNAs are also an important component of the BCL6/GH feedback loop and this regulatory system may contribute to maintaining lipolysis of fat stores at a sufficient level to meet the energy expenditures associated with hatching.

## 5. Conclusions

The metabolic state is a fluidic balance of complex and interconnected feedback loops which respond to hormonal, metabolic, and transcriptional regulatory cues induced by ever-changing environmental and/or nutritional conditions. Disturbing this balance often leads to detrimental consequences for an organism. Therefore, as miRNA are known to mute gene expression rather than ablate it entirely, it is not surprising that they are critical regulators of eukaryotic metabolic processes (reviewed by [51]). Inducing changes in a small subset of miRNAs can quickly alter many metabolic processes concurrently, in response to fluctuations in environmental/nutritional statuses. Though adipocytic miRNA have been relatively well studied in mammals, current knowledge of chicken adipocytic miRNAs is somewhat limited, particularly during the transition from embryonic to post-hatch development. The present study combined miRNome and transcriptome data to identify miRNAs regulating adipose development and function during the chick peri-hatching period. A major metabolic shift occurs during this time, as the newly hatched chick switches from a lipid-rich (yolk) to a lipid poor (feed) energy source. From adipocytic miRNome and transcriptome network mapping, a complex but interconnect regulatory system emerges, which appears to favor lipolytic processes or lipid storage during the embryonic to hatch transition, likely to ensure energy availability to the newly hatched chick in response to the energy expenditures of hatching and increased activity (Figure 8). This regulatory system consists of both master transcriptional regulatory pathways, such as circadian rhythm signaling and GH signaling, as well as a group of miRNAs which work in concert with them to maintain a favorable balance of these regulatory systems during this critical developmental period.

## Figures and Tables

**Table 1:** Top cellular pathways and functions associated with chicken adipose tissue during the embryonic to hatch transition.

Time Point Comparison	Top Canonical Pathways	Top Upstream Regulators	Molecular and Cellular Functions	Physiological System Development and Function
E18-E20	EIF2 signaling; NRF2-mediated oxidative stress response; VEGF signaling; PDGF signaling; IGF-1 signaling	TP53; TGFB1; dexamethasone; β-estradiol; sirolimus	cell death and survival; cellular assembly and organization; cellular function and maintenance; cellular movement; cellular compromise	organismal survival; skeletal and muscular system development and function; organismal development; tissue development
E20-D0	NRF2-mediated oxidative stress response; EIF2 signaling; ILK signaling; tight junction signaling	KRAS; TGFB1; β-estradiol; dexamethasone; MYC	cell death and survival; cellular compromise; lipid metabolism; small molecular biochemistry; molecular transport	organismal survival; organismal development; skeletal and muscular system development and function; tissue morphology
D0-D1	NRF2-mediated oxidative stress response; PI3K/AKT signaling; IGF-1 signaling; 14-3-3-mediated signaling; cell cycle: G2/M DNA damage checkpoint regulation	TGFB1; β-estradiol; TP53; ERBB2; dexamethasone	cell death and survival; cellular movement; cellular growth and proliferation; cellular development; protein synthesis	organismal survival; organismal development; tissue development
D1-D3	calcium signaling; RhoA signaling; signaling by Rho family GTPases; epithelial adherens junction signaling; regulation of actin-based motility by Rho	dexamethasone; TP53; β-estradiol; TNF; MEF2C	cell death and survival; cellular assembly and organization; cellular movement; lipid metabolism; molecular transport	organismal development; organismal survival; skeletal and muscular system development and function; tissue development

## Data Availability

The data presented in this study are openly available in the NIH Short Read Archive at https://www.ncbi.nlm.nih.gov/sra/PRJNA600484, Bioproject PRJNA600484.

## Supplementary Materials

The following are available online at https://www.mdpi.com/2073-4425/12/2/196/s1, Table S1: Primers used in this study, Table S2: All identified miRNAs, Table S3: All identified transcripts.
